# Metacognitive training in the acute psychiatric care setting: feasibility, acceptability, and safety

**DOI:** 10.3389/fpsyg.2023.1247725

**Published:** 2023-11-29

**Authors:** Rabea Fischer, Matthias Nagel, Daniel Schöttle, Daniel Lüdecke, Franziska Lassay, Steffen Moritz, Jakob Scheunemann

**Affiliations:** ^1^Department of Psychiatry and Psychotherapy, University Medical Center Hamburg-Eppendorf, Hamburg, Germany; ^2^Department of Psychiatry and Psychotherapy, Asklepios Clinic North Wandsbek, Hamburg, Germany; ^3^Department of Psychiatry and Psychotherapy, University Hospital Schleswig-Holstein, Luebeck, Germany; ^4^Department of Psychiatry and Psychotherapy, Asklepios Clinic Harburg, Hamburg, Germany

**Keywords:** locked ward, psychosis, psychiatry, psychological intervention, group therapy, severe mental illness, metacognitive training

## Abstract

Patients on acute psychiatric wards desire more psychosocial treatment than they receive, according to recent studies, but evidence-based interventions tailored to this setting are currently lacking. Metacognitive Training for psychosis (MCT) is a flexible, easy-to-administer group therapy that has been adapted to meet this demand (MCT-Acute). Thirty-seven patients with severe mental illness took part in MCT-Acute twice a week during their stay on a locked acute ward and were interviewed before, during, and after the intervention period regarding subjective utility, subjective adverse events, and symptom severity; attendance rates and reasons for absence were recorded. In addition, staff rated adverse events, symptom severity, and functioning (German Clinical Trial Register ID: DRKS00020551). Overall, most patients evaluated MCT-Acute positively and reported symptom stabilization. Staff also reported improvement in functioning. No clinician-rated adverse events related to participation in MCT-Acute were reported. Conducting MCT-Acute is feasible and safe and may contribute to meeting patients’, practitioners’, and researchers’ demands for more evidence-based psychotherapeutic interventions for the acute psychiatric care setting.

**Clinical Trial Registration:**

ID: DRKS00020551, https://drks.de/search/de/trial/DRKS00020551

## 1 Introduction

Risk of harm to oneself or others represent key aspects of patient safety in inpatient psychiatry (Marcus et al., 2021) and constitute legal grounds for acute involuntary psychiatric inpatient treatment in many parts of the world (Rains et al., 2019; Saya et al., 2019). While 9.1% of all Europeans experience suicidal ideation in their lifetime (Castillejos et al., 2020), this number rises to 34.5% for people diagnosed with schizophrenia (Bai et al., 2021) and individuals with a diagnosis of bipolar disorder show suicide attempt rates at least 20 times higher than the adult general population (Tondo et al., 2021). Patients with schizophrenia or bipolar disorder are also at higher risk of committing crimes (Senior et al., 2020; Yee et al., 2020), although they are overall responsible for only a small fraction of all crimes committed, a much larger number of people experiencing psychosis are victims rather than perpetrators of violent crimes (Thornicroft, 2020). People with psychosis also show victimization rates several times higher than the general population (de Vries et al., 2019). Thus, one essential purpose of acute psychiatric services has been to assess and, where possible, avoid harm, at times placing little emphasis on fostering positive aims through therapeutic means (Bowers et al., 2014; Tracy and Phillips, 2022).

Acute psychiatric care has moved from custodial models of treatment, often meaning indefinite confinement and equating mental illness with criminality, to curative goals, shared decision-making, and increasing attempts to integrate care into the community today (Saya et al., 2019; Johnson et al., 2022). Yet, in many countries, acute psychiatric care still ordinarily takes place in inpatient settings, often on locked wards staffed by a multidisciplinary team of psychiatrists, nurses, and specialized therapists. Even in places where a variety of psychiatric emergency services exist outside of hospitals, such as in the United Kingdom (e.g., Odejimi et al., 2020), psychiatric emergency wards for patients in acute crisis still exist. In many cases, patients are mandated to enter inpatient care, and in some countries they may experience involuntary treatment lasting up to several months (Zhang et al., 2015; Sashidharan et al., 2019). At this stage of treatment, psychological interventions offer a range of benefits such as identifying problems and strategies to reduce them, reducing stress, fostering a recovery-oriented outlook and hope through the therapeutic relationship, improving social functioning and treatment compliance and reducing risk of rehospitalization (Donaghay-Spire et al., 2016; Barnicot et al., 2020).

Psychological care is often lacking during the acute stage, even though many patients endorse more therapeutic interactions with ward staff and several national treatment guidelines for severe mental illnesses explicitly call for psychosocial treatment options across the various stages of the illness, including during the acute phase (National Institute for Health and Care Excellence (NICE), 2014; Wood and Alsawy, 2016; American Psychiatric Association (APA), 2020; Berry et al., 2022). In recent years, several psychological interventions have been developed for the acute care setting. For instance, Jacobsen et al. (2020) examined a mindfulness-based crisis intervention for patients with psychosis. No drop-outs were observed during the intervention, and it was associated with a decreased risk of readmission and relapse rates at 12 months’ follow-up. Paterson et al. (2019) examined a cross-diagnostic psychologically informed acute inpatient therapy service that provided both individual and group sessions, and found that their intervention was feasible to conduct with acute inpatients and that it might lead to reduced psychological distress and increased mental health-related self-efficacy compared to treatment as usual. However, evidence-based interventions specifically designed or adapted to fit this particular setting are scarce and are rarely implemented in the clinical context. Studies evaluating their efficacy are lacking (Paterson et al., 2019; Berry et al., 2022).

Several factors unique to the acute ward setting make the evaluation of such interventions particularly challenging. One of these is the high symptom load, especially neurocognitive impairments, which make it difficult for participants to answer even short and/or simple questionnaires, along with the high distress that participants often experience as a result (Wood et al., 2021; Berry et al., 2022). Accordingly, comprehension is often low and informed consent cannot always be properly obtained. Another characteristic of the acute setting that makes research particularly challenging is that in many countries there is no continuity of treatment from the acute inpatient to subsequent (open) settings (Wood et al., 2022), although care continuity, particularly the ability to build a therapeutic relationship, is associated with a variety of positive outcomes (Ruud and Friis, 2022). As stays on acute wards are often brief, ranging from a few days to around four weeks, and interventions that are limited to the ward itself cannot continue seamlessly once the patient leaves care, interventions must be very brief as well (Bullock et al., 2021). Due to the high turnover of patients, group interventions in particular should not be sequential so that patients can join the intervention at any time point and can resume participation without having missed essential information if they miss sessions due to worsening of symptoms or other reasons (Fife et al., 2019). In addition, it is often difficult to contact participants for follow-up assessments after they have been discharged from the ward (Paterson et al., 2019; Raphael et al., 2021a).

There are also several barriers to the implementation of psychological therapies itself, including the busy ward setting with frequent emergencies and departures from routine treatment, lack of training of ward staff, lack of support from leadership, acute exacerbation of symptoms precluding, for example, the ability to concentrate for several minutes, as well as lack of specific adaptation of interventions to the acute care setting (Evlat et al., 2021; Raphael et al., 2021b).

In order to address the aforementioned challenges and to contribute to narrowing the current treatment gap for patients with acute symptoms, particularly on closed wards, we developed the Metacognitive Training for the acute psychiatric setting (MCT-Acute). The MCT-Acute is an adaption of Metacognitive Training for psychosis (MCT; Moritz and Woodward, 2007a). MCT is a psychological group intervention based on more than 30 years of research suggesting that individuals who experience psychosis are prone to certain cognitive biases that underlie the foundation and maintenance of psychotic symptoms, particularly delusions (e.g., Moritz et al., 2017; Ward and Garety, 2019). One of the most researched biases that constitutes a key mechanism in the development of delusions is the jumping to conclusions bias (Dudley et al., 2016; McLean et al., 2017), in which participants make hasty decisions based on very little information (Garety et al., 1991). Research has also shown that patients with psychosis demonstrate a bias against disconfirmatory evidence (e.g., Woodward et al., 2006; Veckenstedt et al., 2011) and do not revise their decision, even when they are confronted with evidence that goes against their decision. This bias also constitutes a central mechanism in the development and maintenance of delusions (Eisenacher and Zink, 2017). Another cognitive bias contributing to the development of delusions, particularly persecutory delusions (Murphy et al., 2018), is the self-serving attributional style first described by Kaney and Bentall (1989), Bentall et al. (1991, 1994). MCT is a multimedia-based group intervention that uses engaging exercises to provoke, for example, hasty decision making within a group session and thus produce so-called aha moments, allowing patients to recognize their biased thinking directly through the exercise instead of through theoretical explanations. This realization is followed by exercises that help patients develop alternative ways of thinking. According to recent meta-analyses, MCT is effective for a range of symptoms, particularly delusions and positive symptoms overall (Eichner and Berna, 2016; Liu et al., 2018; Sauvé et al., 2020; Penney et al., 2022). However, it is too challenging and difficult for many patients with high symptom severity (van Oosterhout et al., 2014). In addition to MCT for psychosis, versions of Metacognitive Training have been developed for other disorders in recent years, including MCT for depression (Jelinek et al., 2013) and suicidality (Jelinek et al., 2021), depression in later life (Schneider et al., 2018), obsessive-compulsive disorder (Miegel et al., 2022), gambling disorder (Gehlenborg et al., 2021), and borderline personality disorder (Schilling et al., 2018). A case report describes the adaptation process of MCT-Acute in detail and outlines its potential as an add-on treatment in the acute-care setting (Fischer et al., 2022). MCT-Acute was designed to be suitable for patients with psychosis but also for patients with (comorbid) depression. Most topics that are addressed by MCT for psychosis are also relevant to individuals with depression, although the emphasis may differ between psychosis and depression (e.g., self-serving attributional style in psychosis vs. depressive attributional style in depression). In addition, several modules in MCT for psychosis already address depression-specific topics, such as mood and self-esteem. Furthermore, one module was adapted from the MCT for depression; thus, MCT-Acute also targets depression-specific cognitive biases that may be relevant to patients on acute wards with a variety of primary diagnoses who suffer from (comorbid) depression.

The aim of the present feasibility trial was to assess the acceptability and safety of the adapted version of a well-researched, easy-to-implement, evidence-based intervention. In particular, we aimed to assess whether patients on acute psychiatric wards who are being treated for different forms of severe mental illness (mainly psychosis but also depression, borderline personality disorder, and substance use disorder) would attend the offered sessions (and why they would not), whether they would view the treatment as useful, and whether they would experience any adverse events or symptom worsening related to their participation. Regarding safety, we not only assessed adverse events rated by clinical staff but also included subjective adverse events as side effects occur not only with pharmacological treatment but also with psychotherapy (Linden and Schermuly-Haupt, 2014). Thus, the pilot trial addressed the following hypotheses. We hypothesized that patients in an acute psychiatric inpatient setting would be willing to attend MCT-Acute sessions, that they would rate MCT-Acute as subjectively useful, and that there would be no severe subjective adverse events or unwanted events associated with participation in MCT-Acute. In addition, we hypothesized that patients’ clinician-rated and self-rated overall symptom severity would decrease significantly and that patients’ overall functioning would increase significantly over the course of the intervention period.

## 2 Materials and methods

### 2.1 Design

The trial was planned as an uncontrolled, observational pilot trial that included patients with severe mental disorders in an acute locked psychiatric setting. We decided against a controlled trial because a wait-list control design would not be feasible in this setting and there was no suitable control group program for this setting available. In addition, the trial’s primary aim was to prove the feasibility and safety of the intervention. Patients could attend MCT-Acute sessions over a period of 3.5 weeks in addition to a standardized acute inpatient treatment program (including, e.g., psychopharmacotherapy and occupational therapy). Before the first group session (t0; baseline assessment), after two weeks of intervention (t1; interim assessment) and after four weeks of intervention (t2; post assessment), participants completed clinical interviews comprising self- and other-rated symptom assessments as well as questionnaires regarding the subjective utility and subjective adverse events of the intervention. Prior to their participation, all patients gave written informed consent. The University Medical Center Hamburg-Eppendorf’s Ethics Committee for Psychological Studies approved the study (LPEK-0108); we preregistered the study in the German Clinical Trial Register (DRKS-ID: DRKS00020551). The preregistration included further measures that will be reported elsewhere as they do not immediately relate to the feasibility and safety of the intervention.

### 2.2 Setting

The trial was conducted at two sites: the Department of Psychiatry and Psychotherapy of the University Medical Center Hamburg-Eppendorf and the Department of Psychiatry and Psychotherapy of the Asklepios Clinic Hamburg North (both in Germany). The University Medical Center Hamburg-Eppendorf includes two locked inpatient units (crisis intervention wards) with 13 and 19 beds, respectively. The Department of Psychiatry and Psychotherapy of the Asklepios Clinic North also includes two locked inpatient units, each with 21 beds. The hospitals’ catchment areas are urban areas with approximately 450,000 and 320,000 residents, respectively. All four locked acute inpatient psychiatric wards provide care for people with any psychiatric diagnosis that require intensive care to prevent harm, including suicidality or risk of aggression against others.

### 2.3 Sample

Patients were eligible for participation if they had a primary diagnosis of a severe mental disorder (diagnoses classified in the DSM-V or the ICD-10 F-codes), were expected to stay on the ward for at least two weeks, and were at least 18 years old. Exclusion criteria were insufficient command of the German language, intellectual disability, dementia, or inability to confirm consent with a legal guardian where applicable. Patients who were acutely intoxicated were not approached for participation until their intoxication had subsided. Patients admitted to one of the locked wards were screened soon after admission to determine whether they met the inclusion criteria, and eligible patients were approached by study staff regarding trial participation.

**Figure 1:**
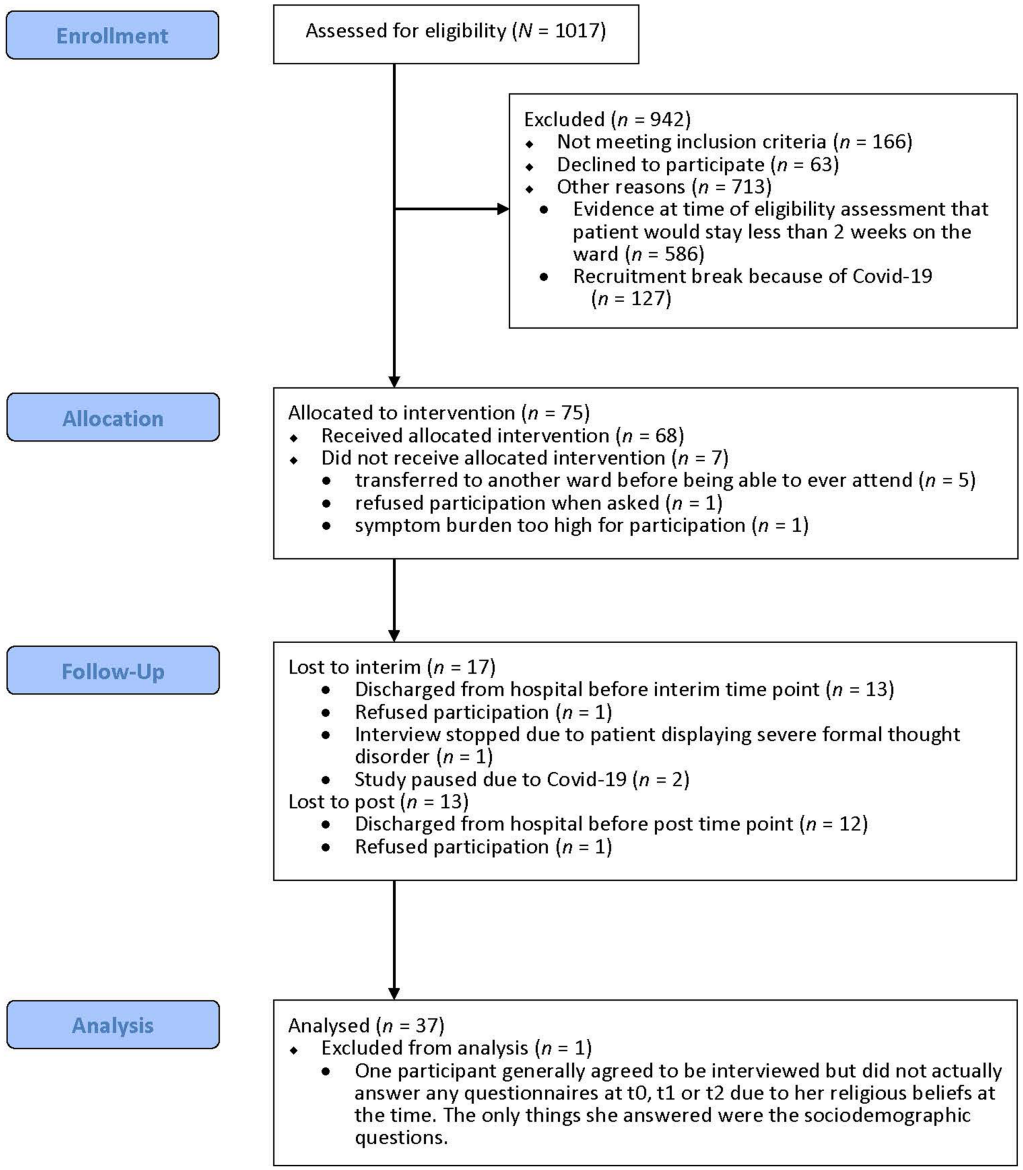
Flow diagram of participant inclusion.

All patients received acute psychiatric standard treatment, including primarily psychopharmacotherapy (all participants were taking psychotropic medication; all but one [2.7%] were taking antipsychotic medication), as well as occupational and physical therapy, doctor’s visits three times per week, one-on-one meetings with a psychologist up to twice a week for some patients, and, at one of the hospitals, psychologist-led group interventions. Additionally, patients were offered the opportunity to take part in MCT-Acute up to two times per week (regardless of their participation in the study). We screened 1017 patients for participation and approached 138, 63 of whom declined participation and 75 of whom were assessed at baseline (see Figure 1). Of those assessed at baseline, seven patients did not participate in any MCT-Acute session. Of the remaining participants, 51 (75.0%) completed the assessment at two weeks and 38 (55.9%) also completed the assessment at four weeks. Whenever participants were unable to complete questionnaires themselves (e.g., due to difficulties concentrating or writing or due to circumstances such as lacking appropriate eyeglasses), they received support from the assessors (e.g., reading questions aloud, writing down participants’ answers). Some participants were unable to complete all questionnaires, due, for example, to high symptom load or poor neurocognitive abilities.

### 2.4 Procedure

#### 2.4.1 Intervention (MCT-Acute)

Two trainers delivered MCT-Acute on the locked acute wards of the two hospitals. Most trainers in this study were psychologists who had completed a master’s degree and were currently undergoing postgraduate training in cognitive behavioral therapy; the other trainers were occupational therapists who worked on the respective wards. At least one psychologist was present during all sessions. Either RF or JS, the developers of MCT-Acute, was present at the majority of the training sessions (*n* = 236, 90.0% of all sessions). RF and JS both received training on MCT’s delivery by its developer SM and have several years of experience delivering MCT for psychosis. All other therapists involved underwent the online training for MCT for psychosis offered by MCT’s developers (see www.uke.de/e-mct) and received intensive one-on-one training by RF or JS. The training took place twice a week. Group size varied between two and nine patients. One cycle through all seven modules of MCT-Acute took 3.5 weeks to complete, although participants could continue participating after they had completed one cycle. The seven MCT-Acute modules address the following topics: *empathy, mood, attributional style, stigma, jumping to conclusions, coping strategies,* and *self-esteem*. We describe the adaptation process from the regular MCT for psychosis (Moritz and Woodward, 2007b) to MCT-Acute in detail in Fischer et al. (2022). All training material can be downloaded free of charge from www.uke.de/mct-acute.

#### 2.4.2 Recruitment

Participants were consecutively recruited shortly after their admission to a locked inpatient ward. In addition to acute psychiatric standard treatment, they were invited to take part in MCT-Acute up to two times per week.

Patients provided written informed consent to participate in the study and then completed the baseline assessment (t0), the interim assessment two weeks later (t1), and the post assessment another two weeks later (t2). In addition, subjective utility, motivation to continue participation, and potentially negative events occurring during the sessions were assessed at the end of each session via a short, non-mandatory questionnaire (Post-Session Questionnaire).

### 2.5 Instruments

#### 2.5.1 Acceptability of the intervention

We determined acceptance and feasibility of the intervention based on the number of attended sessions, reasons for missing sessions, and several feedback questionnaires regarding the intervention.

##### 2.5.1.1 MCT-Acute feedback questionnaire

The MCT-Acute Feedback Questionnaire is based on a questionnaire previously used to evaluate MCT (Moritz and Woodward, 2007a,b). It is designed to capture general feelings, evaluations, and understanding of the participants regarding the MCT-Acute (e.g., *“The MCT-Acute was useful and sensible”*). The present version of the questionnaire comprises 12 quantitative items rated on a four-point Likert scale ranging from 0 (“I do not agree at all”) to 3 (“I agree completely”) and three open-ended items (see Appendix A1). It was administered at t1 and at t2.

##### 2.5.1.2 Session-specific feedback

In addition to administering the feedback questionnaire at t1 and t2, we collected feedback at the end of each session using a brief 10-item questionnaire that included items such as “*MCT-Acute was fun*” and “*MCT-Acute confuses me*.” The first seven items were answered on a three-point scale (from “rather agree” to “rather disagree”), while the last three items were open-ended (see Appendix A2). This questionnaire was handed out not only to study participants but also to other patients who attended the MCT-Acute group and agreed to give anonymous feedback.

#### 2.5.2 Safety

##### 2.5.2.1 Adapted questionnaire about side effects psychosis and internet

The Adapted-QueSPI (based on Rüegg et al., 2018) was adapted to assess potential subjective adverse events of the MCT-Acute at t1 and t2. After removal of items that were inappropriate for the current trial (e.g., “*I experienced technical difficulties that bothered me*”), the questionnaire comprised 13 quantitative items rated on a four-point Likert scale ranging from 0 (“I do not agree at all”) to 3 (“I agree completely”) as well as three open-ended items (see Appendix A3).

##### 2.5.2.2 Unwanted events

Based on the Unwanted Events-Adverse Treatment Reactions Checklist (UE-ATR Checklist; Linden, 2013), we monitored the following unwanted events throughout the intervention period: prolongation of treatment, emergence of new symptoms, deterioration of symptoms, and strains in the patient-therapist relationship. We also monitored suicidal ideation and suicide attempts. We used the UE-ATR Checklist’s relation to treatment rating scheme (1 = “unrelated to therapy,” 5 = “extremely likely due to therapy”), but omitted the context of development and the severity ratings. We based ratings on the ward staff’s clinical documentation of the patients’ behavior on the ward.

#### 2.5.3 Symptoms

We assessed patients’ baseline psychopathology levels and monitored their symptom development throughout the intervention period to detect changes in symptoms across patients.

##### 2.5.3.1 Brief psychiatric rating scale (4.0) expanded version

To assess baseline symptom levels, we administered the BPRS-E (Lukoff et al., 1986; Ventura et al., 1993) at t0, which is comprised of 24 items assessing the presence and severity of a variety of psychiatric symptoms. Its scale points range from 1 (“not present”) to 7 (“extremely severe”), yielding sum scores between 24 and 168 with higher scores indicating more severe psychopathology.

##### 2.5.3.2 Clinical global impressions scale

The CGI (Guy, 1976) is a clinician-rated scale that consists of a Severity (CGI-S) and an Improvement (CGI-I) scale. In the present study, the patient’s treating psychiatrist or the head psychiatrist on the locked ward rated the CGI. The CGI-S reflects the clinician’s assessment of the patient’s present illness status in comparison with other patients from the same clinical population. The CGI-I assesses the improvement or worsening of the patient’s condition since the previous rating. The CGI-S ranges in scores from 1 (“normal, not at all ill”) to 7 (“among the most extremely ill patients”); the CGI-I ranges from 1 (“very much improved”) to 7 (“very much worse”).

##### 2.5.3.3 Brief symptom inventory-18

The BSI-18 (German version: Spitzer et al., 2011) is a short form scale of the Symptom Checklist-90-Revised that measures psychological stress symptoms during the past seven days. The inventory consists of 18 items that assess the three symptom subscales Somatization, Depression, and Anxiety. Each item is rated on a five-point Likert scale (0 = “not at all”; 4 = “extremely”) based on patient reports.

##### 2.5.3.4 Global assessment of functioning scale

The DSM-IV Axis V (GAF; American Psychiatric Association, 2000) assesses overall functioning on a scale from 100 (“superior functioning, no symptoms”) to 1 (“extreme impairment”).

### 2.6 Data analysis

As specified in the preregistration, only participants who had completed assessments at all three time points and who had participated in the intervention at least once (‘completers’) were considered for the final analysis (*N* = 37).

Measurement point t1 mainly served to ensure the presence of at least preliminary data in case too many included patients transferred out of the ward before the post-intervention measurement point t2. Thus, as subjective utility and subjective adverse events at t2 are based on more attended sessions than at t1 for many participants, we report here only the subjective utility ratings and subjective adverse events for t2. Ratings at t1 can be found in Appendices A4 and A5. For subjective utility and subjective adverse events, we focus here on the quantitative data (readers interested in the analysis of the qualitative data may contact the first author).

Clinician-rated symptoms and functioning were assessed by the acute ward’s head physician or the patient’s primary treating physician on the acute ward. Thus, whenever patients transferred to another ward or were discharged from the hospital entirely before t1 or t2, there were no CGI and GAF ratings available for t1 and/or t2. The GAF analysis was run twice; once using only the available data and once using the last observation carried forward method for data imputation.

To assess the acceptability and safety of the intervention, the number of attended sessions, subjective utility, session specific feedback and unwanted events were analyzed descriptively. Symptom improvement was analyzed both descriptively (CGI) and using repeated measures ANOVAs to assess significant changes in patient-rated symptoms (BSI-18) and clinician-rated overall functioning (GAF) over the course of study participation.

## 3 Results

As shown in Table 1, there was no statistically significant difference between completers vs. non-completers (patients who were assessed at t0 but did not complete all three assessments and/or did not participate in the intervention at least once) on any sociodemographic variable (all *p* > 0.1).

**Table 1:** Comparison between patients who were included in the final analysis (completers) and those who were not (non-completers).

	Completers (*n* = 37)	Non-completers (*n* = 38)	
	*M (SD)*	*M (SD)*	Statistics
Age	39.5 (14.0)	38.5 (11.8)	*t* (73) = 0.34, *p* = 0.735, *d* = 0.079
Primary education in years	11.1 (1.6)	10.7 (2.5)	*t* (72) = 0.86, *p* = 0.394, *d* = 0.200
BPRS baseline score	59.6 (19.9)	57.9 (13.5)	*t* (56.456) = 0.40, *p* = 0.692, *d* = 0.099
GAF baseline score	37.5 (9.1)	41.1 (10.9)	*t* (55) = 1.39, *p* = 0.170, *d* = 0.259
BSI-18 baseline score	19.6 (15.0)	15.2 (13.0)	*t* (63) = 1.27, *p* = 0.208, *d* = 0.315
	*n* (%)	*n* (%)	
Gender (female)	17 (45.9)	19 (50)	*χ*^2^ (1, *N* = 75) = 0.12, *p* = 0.725, *V* = 0.041
*Primary diagnosis*			
Mental disorders due to a general medical condition	0	2 (5.3)	-
Substance-Related and Addictive Disorders	2 (5.4)	2 (5.3)	-
Schizophrenia Spectrum and other Psychotic Disorders	26 (70.3)	25 (65.8)	-
Bipolar and Related Disorders	6 (16.2)	8 (21.0)	-
Depressive Disorders	0	1 (2.6)	-
Trauma- and Stressor-Related Disorders	1 (2.7)	0	-
Personality Disorders	2 (5.4)	0	-
*Number of previous admissions*	*n* = 35	*n* = 36	-
0	3 (8.6)	6 (16.7)	*χ*^2^ (2, *N* = 71) = 3.31, *p* = 0.191, *V* = 0.216
1 to 5	22 (62.9)	15 (41.7)	
6 or more	10 (28.6)	15 (41.7)	
*Legal status of stay*			
Voluntary	8 (21.6)	6 (15.8)	*χ*^2^ (2, *N* = 75) = 0.52, *p* = 0.773, *V* = 0.083
Emergency mandatory admission	17 (45.9)	20 (52.6)	
Mandatory admission by legal guardian	12 (32.4)	12 (31.6)	

### 3.1 Acceptability of the intervention

#### 3.1.1 Number of attended sessions and reasons for missing sessions

During their intervention period, participants could attend a maximum of seven sessions of MCT-Acute. On average, patients attended 3.6 sessions (*SD* = 1.85, range 1–7). Of the 259 total sessions, 133 were missed (51.4%). The reasons for missing sessions included participants being discharged from the ward (*X* = 57, 42.9%), declining participation in the session (*X* = 40, 30.1%), currently undergoing seclusion or restraint measures (*X* = 12, 9.0%), being asleep (*X* = 11, 8.3%), other appointments during a given session (*X* = 9, 6.8%), and being judged ineligible by staff for a given session due to acutely high symptomatology (e.g., severe agitation, disorganization; *X* = 4, 3.0%).

#### 3.1.2 Subjective utility

**Figure 2:**
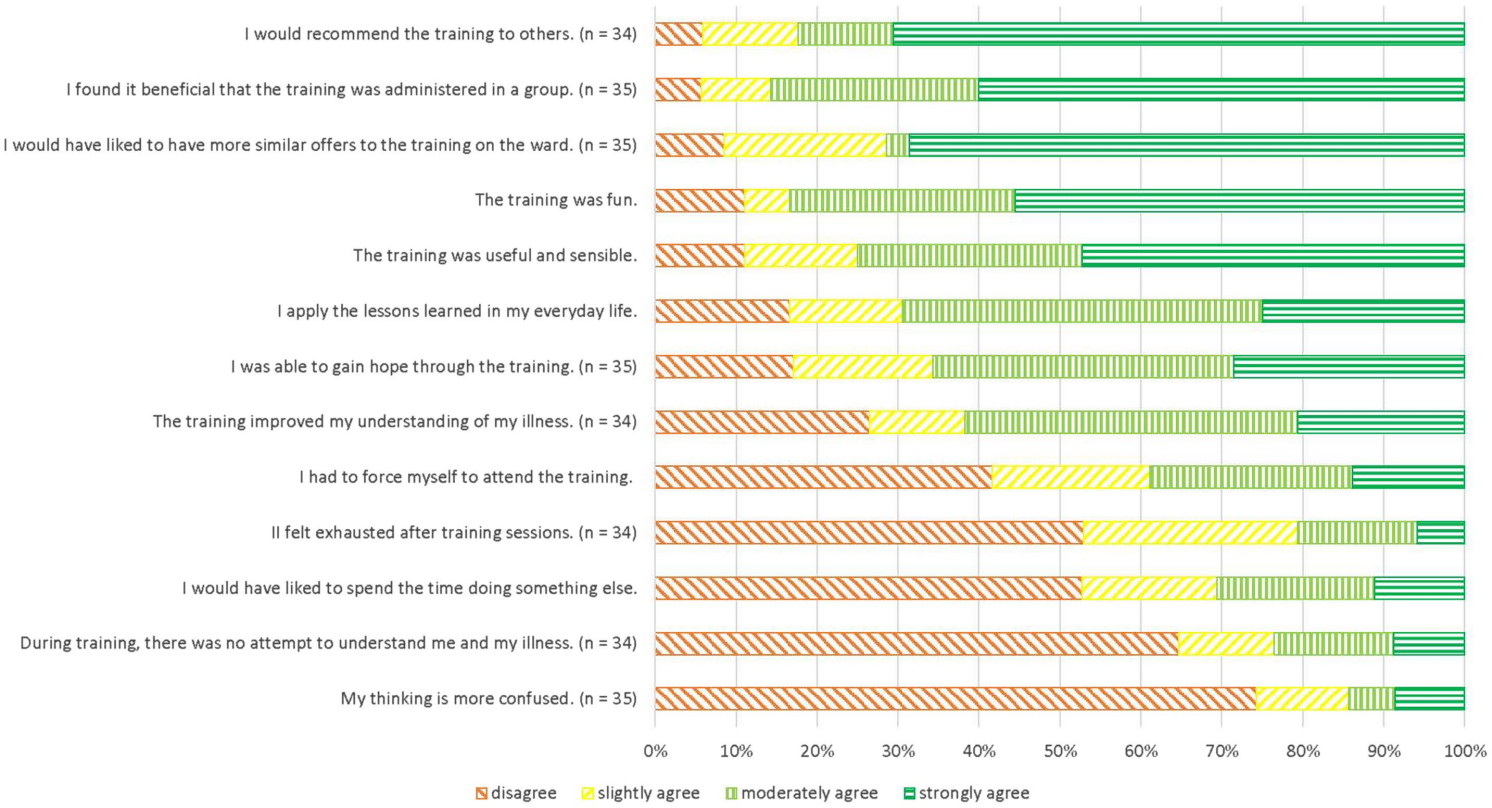
Acceptability of MCT-Acute at t2 in descending order of agreement.

Figure 2 shows participants’ ratings of subjective utility at t2. Overall, participants reported mostly positive experiences with MCT-Acute; a majority fully endorsed that they would recommend MCT-Acute to others (64.9%; *n* = 24) and that they would have liked to have similar interventions to MCT-Acute on the ward (64.9%; *n* = 24). The majority of participants also disagreed with the statement “*My thinking is more confused*” (70.3%; *n* = 26). Subjective utility showed a large negative correlation with subjective adverse events related to the intervention (*r* = −0.67, *p* < 0.001, 95% CI [−0.83, −0.41]).

#### 3.1.3 Session-specific feedback

Of those who attended a given module, 13.6% (*n* = 3; module 7) to 36.4% (*n* = 12; module 4) filled in a questionnaire at the end of the session. Across modules, most participants evaluated the sessions positively, largely rejecting the statement “*MCT-Acute confuses me*” (*X* = 51, 73.9%) and endorsing statements such as “*MCT-Acute was fun*” (*X* = 61, 89.7%; see Table 2). Specifically, only three individual participants endorsed the statement “*MCT-Acute confuses me*” (eight times in total across all modules). Internal consistency of the questionnaire using Cronbach’s alpha was α = 0.55.

**Table 2:** End-of-session feedback summarized over all modules.

	Rather agree (%)	Neither agree nor disagree (%)	Rather disagree (%)	*n*
MCT-Acute was fun.	61 (89.7)	4 (5.9)	3 (4.4)	68
I am motivated to continue participating in MCT-Acute.	60 (87.0)	7 (10.1)	2 (2.9)	69
MCT-Acute helps me.	55 (83.3)	9 (13.6)	2 (3.0)	66
I learned something new during MCT-Acute.	51 (76.1)	10 (14.9)	6 (9.0)	67
MCT-Acute gives me hope for the future.	46 (69.7)	17 (25.8)	3 (4.5)	66
MCT-Acute reduces my health complaints.	35 (54.7)	22 (34.4)	7 (10.9)	64
MCT-Acute confuses me.	8 (11.6)	10 (14.5)	51 (73.9)	69

### 3.2 Safety

#### 3.2.1 Subjective adverse events during MCT-Acute (adapted-QueSPI; self-rating)

Mean endorsements of subjective adverse events did not significantly differ between t1 and t2. The number of subjective adverse events reported at t2 was available for 31 participants and ranged from zero (*n* = 5, 13.5%) to 12 (*n* = 1, 2.7%); on average, participants endorsed 3.1 subjective adverse events (*SD* = 3.03; median = 2). Table 3 shows how many participants endorsed each event. To varying degrees, participants most frequently critically appraised MCT-Acute for not sufficiently considering their personal needs or preferences (54.1%; *n* = 20), and because, after participation in MCT-Acute, they believed that taking medication was less important than they had previously thought (40.5%; *n* = 15). Internal consistency was good (α = 0.82).

**Table 3:** Self-rated side effects at post intervention (t2).

Item	*M* (*SD*)	I do not agree at all (%)	I slightly agree (%)	I moderately agree (%)	I completely agree (%)
MCT-Acute did not sufficiently address my personal needs. (*n* = 35)	1.2 (1.3)	15 (42.9)	7 (20)	3 (8.6)	10 (28.6)
Because of participating in MCT-Acute, I believe that taking medication is less important than I thought before participation. (*n* = 33)	0.9 (1.1)	18 (54.5)	6 (18.2)	4 (12.1)	5 (15.2)
MCT-Acute makes me feel like I am responsible for my problems. (*n* = 35)	0.5 (0.9)	23 (65.7)	6 (17.1)	5 (14.3)	1 (2.9)
My hope of improvement due to MCT-Acute was disappointed. (*n* = 35)	0.6 (1.1)	25 (71.4)	4 (11.4)	1 (2.9)	5 (14.3)
I often did not understand what MCT-Acute tried to tell me. (*n* = 34)	0.5 (1.0)	25 (73.5)	4 (11.8)	2 (5.9)	3 (8.8)
MCT-Acute makes me feel abnormal. (*n* = 33)	0.4 (0.7)	25 (75.8)	5 (15.2)	2 (6.1)	1 (3)
Participation in MCT-Acute reduced my interest to participate in a psychotherapy with personal contact. (*n* = 34)	0.5 (0.9)	26 (76.5)	2 (5.9)	4 (11.8)	2 (5.9)
MCT-Acute overwhelmed me with its abundance of information. (*n* = 35)	0.4 (0.9)	27 (77.1)	3 (8.6)	3 (8.6)	2 (5.7)
I feared that MCT-Acute could increase my symptoms. (*n* = 34)	0.3 (0.8)	28 (82.4)	3 (8.8)	1 (2.9)	2 (5.9)
MCT-Acute has triggered me to lose faith in psychotherapy in general. (*n* = 34)	0.3 (0.7)	28 (82.4)	4 (11.8)	1 (2.9)	1 (2.9)
The participation in MCT-Acute caused me to have more conflicts with others.(*n* = 34)	0.2 (0.6)	28 (82.4)	4 (11.8)	2 (5.9)	0 (0)
The participation in MCT-Acute has put pressure on me. (*n* = 34)	0.2 (0.7)	29 (85.3)	3 (8.8)	1 (2.9)	1 (2.9)

#### 3.2.2 Unwanted events (clinician rating)

Overall, we recorded unwanted events for 17 participants (45.9%), 15 of whom experienced more than one unwanted event. We recorded extension of treatment for 15 patients, worsening of symptoms for nine, emergence of new symptoms for three, and suicidal ideation for one. All of these events (100%) were classified as either unrelated (66.0%) or probably unrelated to the intervention (33.0%).

### 3.3 Symptoms

#### 3.3.1 CGI (clinician rating)

CGI-Severity scores at t0 ranged from moderately ill (*n* = 5; 13.5%), to markedly ill (*n* = 5; 13.5%), to severely ill (*n* = 18; 48.6%), and finally to among the most extremely ill patients (*n* = 6, 16.2%). For three participants (8.1%), there was no CGI-S rating available.

CGI-Improvement ratings at t1 ranged from much improved (*n* = 6; 16.2%), to minimally improved (*n* = 9; 24.3%), to no change (*n* = 14; 37.8%), and finally to minimally worse (*n* = 1; 2.7%). For seven participants (18.9%), there was no CGI-I rating available at t1.

At t2, CGI-I ratings ranged from much improved (*n* = 1; 2.7%) to minimally improved (*n* = 11; 29.7%), to no change (*n* = 9; 24.3%), to minimally worse (*n* = 1; 2.7%), and finally to much worse (*n* = 1; 2.7%). For 14 participants (37.8%), there was no CGI-I rating available at t2.

Two of the participants got worse during their intervention period according to the clinician ratings. The participant whose condition was minimally worse at t1 was also the participant whose condition was much worse at t2. His initial CGI-Severity rating was among the most extremely ill patients. The participant whose condition was minimally worse at t2 had also received an initial CGI-Severity rating of being among the most extremely ill patients. Neither patient’s treating physician attributed their patient’s worsening to their participation in MCT-Acute.

#### 3.3.2 BSI-18 (self-rating)

Numerically, patients improved on the BSI-18 scale from t0 to t2. A repeated measures ANOVA using the Greenhouse–Geisser correction revealed a small sized difference in BSI-18 scores between time points that failed to reach significance (*F*(1.371, 37.013) = 0.49, *p* = 0.546, η_p_^2^ = 0.018). Internal consistency was excellent at all three time points (α_t0_ = 0.91; α_t1_ = 0.91; α_t2_ = 0.94).

#### 3.3.3 GAF (clinician rating)

GAF scores for all three time points were available for 21 of the participants. For these, a repeated measures ANOVA using the Greenhouse–Geisser correction determined that there was a large difference in GAF scores between time points, with scores increasing over time (*F*(1.416, 28.311) = 17.79, *p* < 0.001, η_p_^2^ = 0.471). Using the last observation carried forward method of data imputation, the repeated measures ANOVA using the Greenhouse–Geisser correction still found a large increase in GAF scores over time (*F*(1.332, 47.943) = 20.44, *p* < 0.001, η_p_^2^ = 0.362).

### 3.4 Correlations between outcomes

There were no other significant correlations between outcomes (see Appendix A6).

## 4 Discussion

We assessed the feasibility, acceptability and safety of the Metacognitive Training version adapted for the acute inpatient care setting (MCT-Acute). A sample of 37 patients on closed wards, the majority of whom were classified as at least severely ill, were assessed at baseline and then two weeks and four weeks later. Participants evaluated MCT-Acute positively, the majority stating that they would recommend the training to others and that they would have liked more therapeutic interventions similar to it offered on the ward. Negative subjective evaluations mostly concerned MCT-Acute not addressing participants’ individual needs sufficiently. As symptoms decreased across the sample throughout the intervention period, we deem the intervention safe for application in the acute ward setting.

Overall, patients took part in about half of the sessions they could have attended during their intervention period, resulting in an average of three attended sessions per participant, similar to Paterson et al. (2019). The majority of missed sessions in the present study were missed not because of the patients’ direct choice but, for example, because they were released from the ward early (42.9%). Fife et al. (2019) also found discharge from the ward to be the most common reason for not attending their group (45%). In only 15.4% of all sessions, patients directly declined participation in MCT-Acute. Reasons for this included participants not feeling well on a given day, conflicts with other patients who might be attending the group, other appointments (e.g., with a social worker), or visits from family and were similar to those described in other interventions in the acute setting (e.g., Heriot-Maitland et al., 2014; Fife et al., 2019).

The subjective utility of MCT-Acute was high and comparable to that of Metacognitive Training for patients with psychosis (Moritz and Woodward, 2007b) and of MCT for other disorders such as depression (Jelinek et al., 2017) or OCD (Jelinek et al., 2018). What is new about MCT-Acute is that it specifically targets patients who are in a highly acute crisis and/or are experiencing severe symptoms. With this, MCT-Acute aims to fulfill both, patients’ need for more therapeutic interactions (Wood and Alsawy, 2016) as well as researchers’ calls for documenting adaptations of psychological therapies to acute inpatient care (Jacobsen et al., 2020). In particular, the high endorsement of the statement “I would have liked more similar offers to this one on the ward” (64.9%) shows that patients are open to participating in psychological therapies during the acute stage of illness. Patients’ ability to judge an intervention’s usefulness and their ability to participate in it constitutes an important determinant of patient engagement with psycho social interventions (Raphael et al., 2021b). This is an encouraging result for the continued adaptation of evidence-based psychological therapies to the acute setting.

In recent years, several other psychological/non-pharmacological interventions have been developed for the acute setting and examined in clinical trials. These interventions target a variety of therapeutic aims, including reducing specific symptoms such as self-harm or psychotic symptoms as well as targeting dysfunctional processing and high levels of arousal more generally. The interventions also vary regarding their target populations (e.g., patients with psychosis vs. transdiagnostic) and their mode of delivery (individual, group, or combined approaches). For instance, Fife et al. (2019) examined a DBT-based group intervention focused on self-harm and crisis management strategies regarding feasibility. The authors used content analysis to show that their participants viewed the strategies they were taught in the program to be helpful (Fife et al., 2019). Both Paterson et al. (2019) and Bullock et al. (2021) examined therapeutic approaches based on the comprehend, cope and connect approach (CCC; Clarke and Nicholls, 2018), which grants participants the opportunity to express their emotions, understand the context of their current crisis better, and strengthen self-efficacy. Paterson et al. (2019) reported descriptive statistics showing small readmission rate differences between the intervention and a TAU control group and small to moderate differences regarding certain psychological distress and self-efficacy measures post-intervention. Bullock et al. (2021) found significantly increased mood ratings post- vs. pre-intervention as well as a high mean post-intervention helpfulness rating as indicators of acceptability. Trials examining psychosis-specific non-pharmacological interventions in the acute care setting include Jacobsen et al. (2020) who compared a mindfulness-based crisis intervention (MBCI) with an active control condition (social activity therapy). Their main outcome, readmission rate, was similar across groups at 6 months’ follow-up and lower in the intervention group at 12 months’ follow-up. Thus, despite the various challenges to conducting research on non-pharmacological interventions in the acute inpatient psychiatric setting, the body of literature is increasing, particularly within the last few years, and the present trial contributes to building a more solid scientific basis for such interventions.

Concerns that psychosocial interventions may not be sufficiently understood by patients or that they may be too distressing constitute barriers to the implementation of such interventions (Raphael et al., 2021b), so at the end of each session we assessed whether patients were confused by MCT-Acute. Only three participants endorsed feeling confused after one or more sessions of the intervention, with the majority reporting they were able to follow the training. At the same time, in 89.7% of the questionnaires that were completed, participants indicated that the intervention was fun, which is similar to results from other MCT interventions (e.g., Jelinek et al., 2017).

Although the number of subjective adverse events reported ranged from zero to 12, the majority of participants reported 3 or fewer events. The most frequently voiced critique, that MCT-Acute did not sufficiently address a participant’s personal needs or preferences, is a commonly voiced argument against group therapy (Shechtman and Kiezel, 2016). However, some patients also mention that they prefer group therapy because it allows them to share experiences with other group members (Osma et al., 2019). Practitioners agree that establishing a sense of sharing and belonging to a collective, as well as learning from other participants, are among the key advantages of the group setting which may outweigh drawbacks such as the inevitable lack of individualization (Kealy and Kongerslev, 2022) and lack of privacy as well as participants’ fear of criticism from others (Osma et al., 2019; Raphael et al., 2021b).

The definition of unwanted events and whether they involve statements about causality vary considerably across clinical trials, particularly those assessing psychotherapy (Klatte et al., 2022). In trials in the acute setting, adverse events, including events related to investigating psychological therapies and/or the acute setting specifically, are common but mostly occur independent of participation in the investigated intervention (e.g., Paterson et al., 2019; Jacobsen et al., 2020). Thus, the reported adverse events recorded in this trial (e.g., extension of stay, worsening of symptoms) were expected. Importantly, based on the ward staff’s ratings, none of the reported unwanted events were directly associated with participation in MCT-Acute. Similarly, based on the judgment of the ward’s head psychiatrist or the patients’ treating psychiatrist (CGI) only two participants’ conditions became significantly worse during the intervention period; neither of these cases were related to the intervention, in the psychiatrists’ opinion. Similarly, self-rated symptoms and clinician-rated psychosocial functioning improved across patients throughout the intervention period. These results are encouraging as they support the perspective that psychological interventions in the acute setting are not harmful to patients but may, in fact, aid with problem formulation, stress reduction, and fostering hope. (Donaghay-Spire et al., 2016).

### 4.1 Limitations

The present study has several limitations, such as a comparatively small sample size and the high number of patients who dropped out of the study and were therefore not analyzed further. High patient fluctuation and challenges in recruiting acutely ill patients suffering from severe mental illness for studies in acute psychiatric settings are common. For the present study, assessments could still be conducted when patients were transferred to another ward and were even offered online for patients to complete at home after they had been discharged from the hospital. Still, the present sample was most likely skewed toward the more severely ill patients as by far the most frequent reason for dropout was discharge from the hospital due to sufficient stabilization. As many studies have shown the feasibility of Metacognitive Training programs for moderately acutely ill patients, the likely bias within the present sample does not take away from the finding that MCT-Acute is feasible and safe for severely acutely ill patients. Average attendance rates were low for multiple reasons (e.g., being discharged from the ward early) but were comparable to other studies (Paterson et al., 2019). Another limitation is that we did not include a control group, and we assessed transdiagnostic (global) symptom severity rather than disorder-specific symptoms. In addition, based on the study design, we cannot discern the impact that MCT-Acute had on patients’ symptom development as opposed to the impact of the various other therapies that constitute the treatment as usual on acute wards. As assessment of safety rather than symptom improvement was the aim of this study, we can conclude that stabilization and improvement, regardless of underlying causes, constitute a positive outcome. Since the majority of participants had a diagnosis of schizophrenia spectrum disorder or bipolar disorder, the generalizability of our results to other disorders that patients frequently present with on an acute ward, such as depression and borderline personality disorder, is limited. However, there was no indication that MCT-Acute might be less feasible or safe to conduct with patients who suffer from these disorders. This trial demonstrates that MCT-Acute is feasible and safe as well as valued by patients, countering the broad skepticism regarding conducting any type of psychological individual or group therapy with severely acutely ill patients (Evlat et al., 2021; Raphael et al., 2021b).

### 4.2 Clinical implications

MCT-Acute is a highly standardized and easy-to-implement intervention. Our results add to the growing body of literature on psychologically informed interventions for the acute setting that demonstrates the feasibility of specifically tailored, flexibly administered programs that take into account patients’ particular needs during the acute phase. MCT-Acute enables practitioners to deliver an intervention based on well-researched cognitive mechanisms that is well accepted by patients, even during the acute stage of illness.

### 4.3 Future research

Researchers should conduct a larger MCT-Acute trial, including a control group, to examine positive symptoms as well as cognitive bias measures pre and post intervention in order to replicate MCT’s mechanism of action. In order to increase the sample size and to address the number of drop-outs due to discharge from the hospital, future studies should increase efforts to reach patients at the later assessment time points, e.g., by using monetary incentives and by emphasizing the possibility of conducting assessments via phone from home. To recruit more patients with non-psychosis diagnoses, researchers might consider offering participation in MCT-Acute even after patients have left the locked acute ward. This would also attenuate the inherent selection bias toward more severely impaired patients who are likely to stay longer on acute wards.

## 5 Conclusion

Patients experiencing acute exacerbations of mental illness value the opportunity to participate in interventions such as MCT-Acute on their acute psychiatric ward, mirroring prior reports that patients with severe mental illness are open to psychotherapeutic treatment. The lack of evidence-based interventions tailored specifically for this setting, together with our finding that MCT-Acute is acceptable and feasible, demonstrates that more research and efforts should be devoted to the development of psychosocial treatment options during acute mental health crises. As an easy-to-implement, freely available intervention program, MCT-Acute can represent one component of a biopsychosocial treatment plan for patients on acute psychiatric wards.

## Data availability statement

The raw data supporting the conclusions of this article will be made available by the authors, without undue reservation.

## Ethics statement

The studies involving humans were approved by the University Medical Center Hamburg-Eppendorf’s Ethics Committee for Psychological Studies. The studies were conducted in accordance with the local legislation and institutional requirements. The participants provided their written informed consent to participate in this study.

## Author contributions

RF, JS, SM, and MN conceived and planned the project. RF, JS, and FL carried out the study. SM and MN supervised the project. DS and DL helped supervise the project. RF wrote the manuscript with support from JS, SM, MN, DS, DL, and FL. All authors contributed to the article and approved the submitted version.
