# Supplementary material for: Metacognitive training in the acute psychiatric care setting: feasibility, acceptability, and safety
Source: Front Psychol. 2023 Nov 29;14:1247725. doi: 10.3389/fpsyg.2023.1247725 (PMC10718302; doi:10.3389/fpsyg.2023.1247725)
Supplement: Supplementary file 1 [file Table_1.pdf]

Appendices A1-A3: Supplementary Questionnaire Information

A1: MCT-Acute Feedback Questionnaire: Qualitative Items

1. What did you like about MCT-Acute?
2. What did you dislike about MCT-Acute?
3. What would you change about MCT-Acute?

A2: Session-specific feedback: Qualitative Items

1. My takeaway from today's session is:
2. What I liked best about the training:
3. What I disliked about the training:

A3: Quespi Qualitative Items

a. Have new symptoms emerged during the intervention period? If yes, which ones? Do you think those new symptoms emerged because of the MCT-Acute?

b. Did some symptoms get worse during the intervention period? If yes, which ones? Do you think that this is because of the MCT-Acute?

c. Were there certain events which lead to a worsening of symptoms during the intervention period? If yes, which ones? Are those events in any relation to the MCT-Acute?
