# Supplementary material for: Metacognitive training in the acute psychiatric care setting: feasibility, acceptability, and safety
Source: Front Psychol. 2023 Nov 29;14:1247725. doi: 10.3389/fpsyg.2023.1247725 (PMC10718302; doi:10.3389/fpsyg.2023.1247725)
Supplement: Supplementary file 2 [file Table_2.pdf]

Appendix A5: Supplementary Table

Table A5.
Self-rated side effects at t1

Item	M (SD)	I do not agree at all	I slightly agree	I moderately agree	I agree completely
5. Because of participating in MCT-Acute, I believe that taking medication is less important than I thought before participation. (*n* = 31)	0.9 (1.2)	17 (54.8)	5 (16.1)	3 (9.7)	6 (19.4)
4. MCT-Acute did not sufficiently address my personal needs. (*n* = 35)	0.8 (1.1)	20 (57.1)	5 (14.3)	6 (17.1)	4 (11.4)
7. I often did not understand what MCT-Acute tried to tell me. (*n* = 34)	0.7 (1.0)	23 (67.6)	3 (8.8)	5 (14.7)	3 (8.8)
3. Participation in MCT-Acute reduced my interest to participate in a psychotherapy with personal contact. (*n* = 33)	0.4 (0.8)	24 (72.7)	6 (18.2)	1 (3.0)	2 (6.1)
2. MCT-Acute makes me feel like I am responsible for my problems. (*n* = 34)	0.6 (1.1)	26 (76.5)	2 (5.9)	1 (2.9)	5 (14.7)
10. My hope of improvement due to MCT-Acute was disappointed. (*n* = 32)	0.4 (0.9)	26 (81.3)	2 (6.3)	2 (6.3)	2 (6.3)
6. MCT-Acute makes me feel abnormal (*n* = 35)	0.4 (0.9)	27 (77.1)	3 (8.6)	3 (8.6)	2 (5.7)
11. The participation in MCT-Acute has put pressure on me. (*n* = 34)	0.5 (1.0)	27 (79.4)	1 (2.9)	3 (8.8)	3 (8.8)
8. MCT-Acute overwhelmed me with its abundance of information. (*n* = 34)	0.3 (0.7)	27 (79.4)	3 (8.8)	4 (11.8)	0
1. MCT-Acute has triggered me to lose faith in psychotherapy in general. (*n* = 35)	0.2 (0.5)	28 (80.0)	6 (17.1)	1 (2.9)	0
9. I feared that MCT-Acute could increase my symptoms (*n* = 34)	0.3 (0.8)	28 (82.4)	3 (8.8)	1 (2.9)	2 (5.9)
12. The participation in MCT-Acute caused me to have more conflicts with others. (*n* = 33)	0.3 (0.7)	28 (84.8)	2 (6.1)	2 (6.1)	1 (3.0)
