# Supplementary material for: Metacognitive training in the acute psychiatric care setting: feasibility, acceptability, and safety
Source: Front Psychol. 2023 Nov 29;14:1247725. doi: 10.3389/fpsyg.2023.1247725 (PMC10718302; doi:10.3389/fpsyg.2023.1247725)
Supplement: Supplementary file 3 [file Table_3.pdf]

Appendix A6: Supplementary Tables

Table A6.
Correlations between outcomes (t2)

Outcome	1	2	3	4	5	6
1. Number of unattended sessions	–					
2. Subjective utility	.28 [−.07, .58]	–				
3. Adapted-QueSPI	−.35 [−.63, .01]	−.67** [−.83, −.41]	–			
4. Unwanted events	.08 [−.26, .40]	−.06 [−.40, .29]	−.08 [−.42, .28]	–		
5. BSI-18	.04 [−.32, .38]	−.11 [−.46, .27]	.11 [−.28, .46]	−.16 [−.49, .20]	–	
6. GAF	.09 [−.30, .45]	−.21 [−.57, .22]	.14 [−.29, .52]	−.14 [−.50, .26]	−.18 [−.54, .24]	–
