# Supplementary figures and images for: Metacognitive training in the acute psychiatric care setting: feasibility, acceptability, and safety
Source: Front Psychol. 2023 Nov 29;14:1247725. doi: 10.3389/fpsyg.2023.1247725 (PMC10718302; doi:10.3389/fpsyg.2023.1247725)

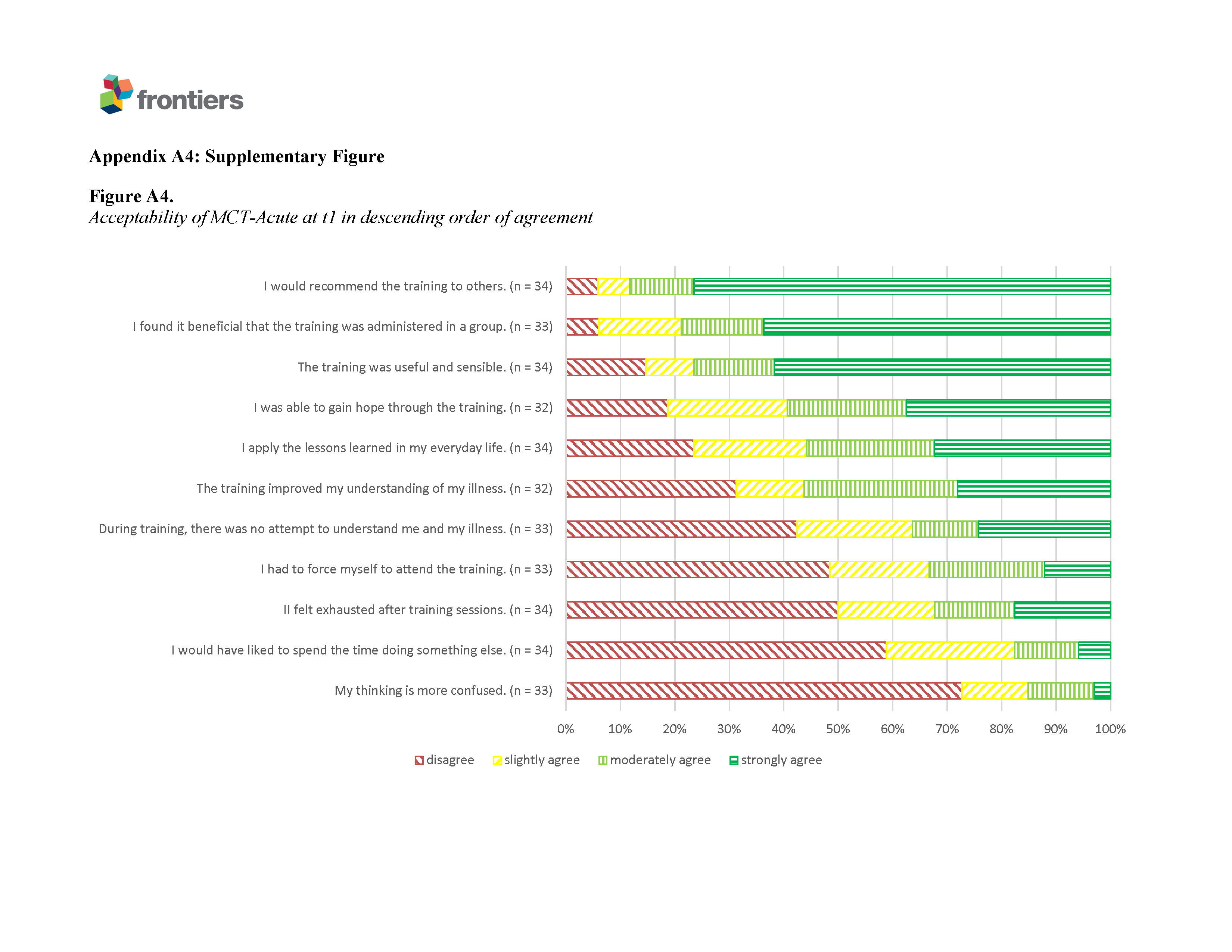

Supplement: Supplementary file 4 [file Image_1.tif]
